# Dose-Dependent Effects of Randomized Intraduodenal Whey-Protein Loads on Glucose, Gut Hormone, and Amino Acid Concentrations in Healthy Older and Younger Men

**DOI:** 10.3390/nu10010078

**Published:** 2018-01-12

**Authors:** Caroline Giezenaar, Natalie D Luscombe-Marsh, Amy T Hutchison, Scott Standfield, Christine Feinle-Bisset, Michael Horowitz, Ian Chapman, Stijn Soenen

**Affiliations:** 1 Discipline of Medicine and National Health and Medical Research Council of Australia (NHMRC) Centre of Research Excellence in Translating Nutritional Science to Good Health, Adelaide Medical School, Adelaide 5000, Australia; caroline.giezenaar@adelaide.edu.au (C.G.); natalie.luscombe-marsh@csiro.au (N.D.L.-M.); amy.hutchsison@adelaide.edu.au (A.T.H.); scott.standfield@adelaide.edu.au (S.S.); christine.feinle@adelaide.edu.au (C.F.-B.); michael.horowitz@adelaide.edu.au (M.H.); ian.chapman@adelaide.edu.au (I.C.); 2 Commonwealth Scientific and Industrial Research Organisation (CSIRO), Food and Nutrition, Adelaide 5000, Australia

**Keywords:** ageing, whey protein, gut hormones

## Abstract

Protein-rich supplements are used widely for the prevention and management of malnutrition in older people. We have reported that healthy older, compared to younger, adults have less suppression of energy intake by whey-protein—effects on appetite-related hormones are unknown. The objective was to determine the effects of intraduodenally administered whey-protein on glucose, gut hormone, and amino acid concentrations, and their relation to subsequent *ad libitum* energy intake at a buffet meal, in healthy older and younger men. Hydrolyzed whey-protein (30 kcal, 90 kcal, and 180 kcal) and a saline control (~0 kcal) were infused intraduodenally for 60 min in 10 younger (19–29 years, 73 ± 2 kg, 22 ± 1 kg/m^2^) and 10 older (68–81 years, 79 ± 2 kg, 26 ± 1 kg/m^2^) healthy men in a randomized, double-blind fashion. Plasma insulin, glucagon, gastric inhibitory peptide (GIP), glucagon-like peptide-1 (GLP-1), peptide tyrosine-tyrosine (PYY), and amino acid concentrations, but not blood glucose, increased, while ghrelin decreased during the whey-protein infusions. Plasma GIP concentrations were greater in older than younger men. Energy intake correlated positively with plasma ghrelin and negatively with insulin, glucagon, GIP, GLP-1, PYY, and amino acids concentrations (*p* < 0.05). In conclusion, intraduodenal whey-protein infusions resulted in increased GIP and comparable ghrelin, insulin, glucagon, GIP, GLP-1, PYY, and amino acid responses in healthy older and younger men, which correlated to subsequent energy intake.

## 1. Introduction

A growing awareness of the extent and adverse effects of aging-related muscle loss, including reduced functional capacity and decreased quality of life [1,2,3], has stimulated the development of nutritional strategies designed specifically to preserve and/or restore skeletal muscle mass and function. A ‘common’ nutritional strategy for management of malnutrition in older people is the use of nutritional supplements, which are usually high-energy drinks rich in whey protein (e.g., 10–30 g protein) [4,5,6]. In younger as well as older adults, higher postprandial plasma amino acid concentrations induce greater muscle protein synthesis [7,8,9], which provides a rationale to increase protein intake in older people. Whey, when compared to casein or soy, protein results in greater muscle protein synthesis in young and older men [8]. Whey protein is a high-quality protein, high in essential, branched-chain amino acids and leucine, which are rapidly digested. Whey ingestion results in post prandial amino acid availability, which stimulates muscle anabolism [10]. It has been suggested that whey protein can be used in a clinical setting to improve health outcomes in those at risk of muscle loss [11].

On the other hand, weight loss and under-nutrition are common in older adults, often associated with, and/or caused by, reduced appetite and food intake. These changes have been termed the ‘anorexia of aging’ [1,3], and may be associated with serious adverse effects. Given that protein is the most satiating macronutrient in young people, and its substitution for other macronutrients is often advocated to promote weight loss in overweight young adults [12,13], the satiating and weight loss-promoting effects of increased protein ingestion could potentially counteract some or all of the muscle benefits of increased protein ingestion in older people. Yet, despite the increasing use of protein-rich drinks by older people, information about their effects on appetite-related gastrointestinal mechanisms in this age group is lacking.

Potential mechanisms involved in the regulation of energy intake include variations in gut hormone release and action (e.g., ghrelin, glucose-dependent insulinotropic polypeptide/gastric inhibiting polypeptide (GIP), glucagon-like polypeptide-1 (GLP-1), and peptide tyrosine tyrosine (PYY)), as well as plasma amino acid concentrations [14,15,16]. We have recently shown that in young men there is a dose-dependent effect of intraduodenal whey-protein infusion on plasma gut hormone [15] and amino acid concentrations [17]. Older, when compared to younger, adults had greater changes in plasma insulin (in response to intraduodenal glucose infusions [18]), GIP (after oral glucose intake [19,20]), and GLP-1 (after oral glucose [20] and mixed macronutrient intakes [21]), but not PYY concentrations (in response to intraduodenal infusions of glucose or lipid [22]). Effects of aging on ghrelin after oral mixed macronutrient intakes were inconsistent [23,24,25,26,27].

Healthy aging is associated with a reduced responsiveness to the suppressive effects of nutrients, including protein, on appetite and energy intake [28,29]. Consistent with this, we have recently demonstrated that suppression of energy intake by protein, ingested either orally (30 g (120 kcal), 70 g (280 kcal) whey-protein loads [30]) or infused directly into the small intestine at rates encompassing the normal rate of gastric emptying of nutrients (0.5 kcal/min (7.5 g, 30 kcal), 1.5 kcal/min (22.5 g, 90 kcal), 3.0 kcal/min (45 g, 180 kcal) [31]), and thereby bypassing ‘oral’ and ‘gastric’ effects, is less in older than younger men.

The aim of the study was to further characterize the effects of intraduodenal whey-protein loads on blood glucose, plasma insulin, glucagon, ghrelin, GIP, GLP-1, PYY, and amino acid concentrations, and their relationships with subsequent *ad libitum* energy intake, in older and younger men. We hypothesized that intraduodenally-administered whey protein would result in load-related responses, related to subsequent energy intake, of glucose, gut hormones, and amino acids, and that these responses would be greater in older than younger men.

## 2. Materials and Methods

### 2.1. Subjects

The pilot study included 10 healthy young men and 10 healthy older men. Our original study characterized the effect of aging on energy intake, perceptions of appetite and gastrointestinal symptoms, and antropyloroduodenal motility in response to infusion of hydrolyzed whey protein directly into the duodenum (thereby bypassing orosensory and gastric factors) at loads lower than (0.5 kcal/min), similar to (1.5 kcal/min), and at the upper end (3 kcal/min) of normal gastric emptying rates (1–4 kcal/min), and a saline control, at a rate of 4 mL/min for 60 min (previously published [31]). We have now further characterized the effects of these infusions on blood glucose, plasma insulin, glucagon, ghrelin, GIP, GLP-1, PYY, and amino acid concentrations, and their relationships with other measures, including with subsequent energy intake. The Royal Adelaide Hospital Research Ethics Committee approved the study protocol (approval ID: 120504, approval date: 1 May 2012), and the study was registered as a clinical trial with the Australia and New Zealand Clinical Trial Registry (www.anzctr.org.au, ACTRN12612000906853). All subjects provided written informed consent prior to their inclusion in the study.

### 2.2. Protocol

Each subject was studied on 4 occasions, separated by 7–14 days, in randomized order. The protocol was described in detail previously [31].

Subjects were provided with a standardized evening meal (beef lasagna (McCain Foods, Wendouree, VIC, Australia), ~591 kcal) to consume on the night before each study, and were instructed to fast overnight from solids and liquids and to refrain from strenuous physical activity until they attended the laboratory at the University of Adelaide, Discipline of Medicine, Royal Adelaide Hospital, at ~08:30 a.m. On arrival, a small-diameter (3.5 mm) catheter (total length: 100 cm, Dentsleeve International, Mui Scientific, Mississauga, ON, Canada) was inserted into the stomach through an anesthetized nostril and allowed to pass into the duodenum by peristalsis. The infusion port of the catheter was located in the proximal small intestine 14.5 cm from the pylorus [31].

**Figure 1:**
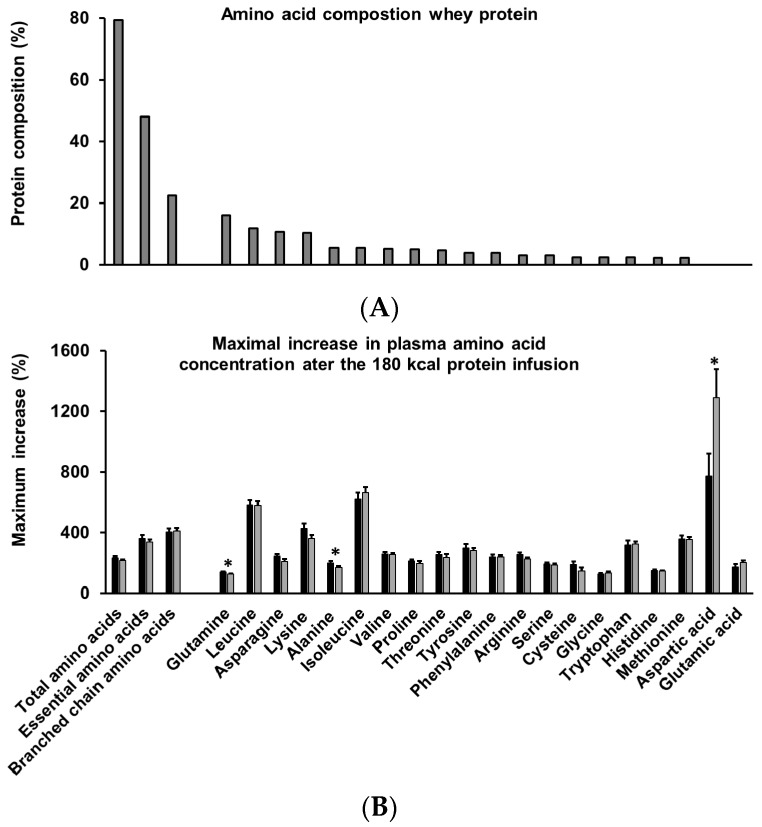
Amino acid composition of the intraduodenally infused whey protein hydrolysate (**A**) and increase of amino acid concentrations at 60 min during the 180 kcal whey protein infusion as a percentage of baseline in healthy younger (*n* = 10 (*n* = 9 for histidine); black bars) and older (*n* = 10; grey bars) men (**B**), ranked in order of high to low amino acid content (g/100 g). Essential amino acids: histidine, isoleucine, leucine, lysine, methionine, phenylalanine, threonine, tryptophan, and valine. Branched-chain amino acids: isoleucine, leucine, and valine. Differences between older and younger men were determined using an independent sample *t*-test. Statistical significance was accepted at *p* < 0.05. * *p* < 0.05 older compared to younger men.

The protein solutions were prepared by dissolving whey protein hydrolysate powder (18.1% Hydrolyzed Whey Protein 821, Fonterra Co-Operative Group Ltd., Palmerston North, New Zealand) in varying amounts of saline and water to achieve the desired loads (i.e., 0.5, 1.5, and 3 kcal/min, which equates to 30, 90, and 180 kcal or 8, 24, and 48 g protein) and to ensure that they were iso-osmotic (640–680 mOsmol/L). The infusions were administered at a rate of 4 mL/min (240 mL over 60 min). The amino acid content of the hydrolyzed (resembling partially digested protein) whey protein is presented in Figure 1.

Immediately before and at 15 min intervals during the intraduodenal infusion, blood samples (an intravenous cannula was positioned intravenously in the right forearm) for measurement of glucose, insulin, glucagon, ghrelin, GIP, GLP-1, PYY, and amino acids were taken (0, 15, 30, 45, and 60 min). Blood samples were collected into ice-chilled ethylenediaminetetraacetic acid (EDTA)-coated tubes. Plasma was obtained by centrifugation for 15 min at 3200 rpm at 4 °C and stored at −80 °C for further analysis.

### 2.3. Measurements

Blood glucose concentrations (millimoles per liter) were determined immediately after collection by the glucose oxidase method using a portable glucometer (Optium Xceed, Abbott Laboratories, Doncaster, VIC, Australia).

Total plasma insulin concentration (milliunits per liter) was measured by Enzyme-linked immunosorbent assay (ELISA) immunoassay (10-1113; Mercodia, Uppsala, Sweden). The minimum detectable limit was 1.0 mU/L. Intra- and inter-assay coefficients of variation were 3.1 and 9.4%. Homeostatic model assessment (HOMA) index at baseline was calculated according to the following formula: insulin concentration (microunits per liter) × glucose concentration (nanomoles per liter)/22.5 [32].

Total plasma glucagon concentration (picograms per milliliter) was measured by RIA (GL-32K; Millipore, Billerica, MA, USA). The minimum detectable limit was 20 pg/mL. The intra- and inter-assay coefficients of variation were 4.4 and 6.3%. The ratio of insulin to glucagon was calculated for each time point in each subject [33].

Plasma total ghrelin concentration (picograms per milliliter) was measured using a radioimmunoassay (RIA) with modifications to the previously published method [34]. The radiolabel was supplied by Perkin Elmer (NEX388; Boston, MA, USA). The standard and samples were incubated with the antibody and radiolabel for 3–4 days at 4 °C. The detection limit was 40 pg/mL. Intra- and inter-assay coefficients of variation were 5.0 and 12.8%.

Total plasma GIP concentration (picomoles per liter) was measured by RIA with modifications to a previously published method [35]. The standard curve was prepared in buffer rather than extracted charcoal stripped serum and the radio-iodinated label was supplied by Perkin Elmer (Boston, MA, USA). The minimum detectable limit was 2 pmol/L. The intra- and inter-assay coefficients of variance were 5.2 and 8.8%. GIP data are not available for one of the younger men.

Total plasma GLP-1 concentration (picomoles per liter) was measured by RIA (GLPIT-36HK; Millipore, Billerica, MA, USA). The detection limit was 3 pmol/L. Intra- and inter-assay coefficients of variance were 6.4 and 9.5%.

Plasma total PYY concentration (picomoles per liter) was measured using RIA (kindly donated by Dr. B Otto, Medizinische Klinik, Klinikum Innenstadt, University of Munich, Munich, Germany) against human peptide YY (1–36) (Sigma-Aldrich, St Lois, MO, USA) and raised in rabbits [15,36]. This antiserum showed <0.001% cross reactivity with human pancreatic polypeptide and 0.0025% cross reactivity with human neuropeptide Y. Standards (1.6–50 fmol/tube) or samples (200 µL plasma) were incubated in 200 µL assay buffer (50 mM NaPO_4_, 10 mM EDTA, 2 g/L gelatin, 0.1 g/L Na-Azide, pH = 7.4) and a 1/12,000 dilution of antiserum for 24 h. The standards and samples were further incubated with 10,000 counts per minute tracer (Perkin Elmer (NEX3410; Boston, MA, USA)) for 24 h. Separation of the antibody-bound tracer from free tracer was by second antibody precipitation (i.e., 500 µL of 1/100 dilution of sheep anti-rabbit immunoglobulin in wash buffer comprising 50 mM Tris-base, 150 mM NaCl, 8% Polyethylene Glycol 6000, pH = 8.0 (Merck KGaA, Darmstadt, Germany), and 50 µL of normal rabbit serum diluted 1/50 in wash buffer), incubated 2 h at room temperature then spun at 4000 rpm at 4 °C for at least 20 min, with supernatants poured off and pellets counted in a gamma counter. The detection limit was 1.5 pmol/L. Intra- and inter-assay coefficients of variations were 8.4 and 13.7%.

Plasma free amino acid concentrations (mmol/L) of asparagine, aspartic acid, alanine, arginine, cysteine, glutamine, glutamic acid, glycine, histidine, isoleucine, leucine, lysine, methionine, phenylalanine, proline, serine, threonine, tryptophan, tyrosine, and valine were analyzed using precolumn derivatization with 6-aminoquinolyl-*N*-hydroxysuccimimidyl carbamate (AQC) performed at the Australian Proteome Analysis’s Facility established under the Australian Government’s National Collaborative Research Infrastructure Strategy (NCRIS). The maximum increase in plasma amino acid concentration of the highest protein load (180 kcal; 3 kcal/min for 60 min) at 60 min was calculated as a percentage of the average baseline concentration. The derivatives were separated and quantified by reversed-phase high-performance liquid chromatography (HPLC). The amino acids (with the exception of tryptophan) were detected by fluorescence, whereas tryptophan required UV detection. Before derivatization, 100 μL of plasma samples were diluted 1:1 with internal standard solution (Norvaline) and deproteinized by ultra-filtration through a membrane with 10 kDa nominal molecular weight cutoff (Ultrfree MC with PL-10 membrane, Millipore, Burlington, MA, USA). Amino acids contained in the filtrate (100 μL) were labeled using the Waters AccQTag™ chemistry and analyzed using a Waters Acquity™ UPLC system (Waters Corporation, Milford, MA, USA). Histidine data are not available for one of the younger men.

### 2.4. Data and Statistical Analysis

Statistical analyses were performed using SPSS software (version 21; IBM, Armonk, NY, USA. The main effects of protein-load and age, and their interaction effects on blood glucose and plasma hormone concentrations and plasma amino acid concentrations at baseline (fasting; 0 min), 15 min after starting the infusion, immediately before the meal (60 min), and net area under the curve (AUC; calculated from baseline to 60 min using the trapezoidal rule), were determined using a repeated-measures mixed-effect model, with protein load as the within-subject factor and age as the between-subject factor, including baseline values at each treatment visit as a covariate. Post-hoc comparisons, adjusted for multiple comparisons using Bonferroni’s correction, were performed when there were significant main or interaction effects. Within-subject correlations between energy intake (published previously [31]) and glucose, and gut hormones and amino acid concentrations, were determined by using a general linear model with fixed slope and random intercept [37]. Assumptions of normality were verified for all outcomes before statistical analysis.

The original study [31] was powered to detect a suppression in energy intake by protein compared to control in 10 subjects per group. We calculated that 10 subjects per group would allow detection of differences in area under the curve (AUC) of the orexigenic hormone ghrelin and the anorexigenic hormone GLP-1 [38] within-subjects (protein compared to control) of 400 pg/mL and 9.1 pmol/L and between age groups (older compared to younger) of 1408 pg/mL and 20.5 pmol/L, respectively, with power equal to 0.8, alpha equal to 0.05 and 10% dropouts. Statistical significance was accepted at *p* < 0.05. Data are presented as mean ± SEM unless otherwise stated.

## 3. Results

Body weight did not differ significantly between the younger (mean ± SD (range): age 23 ± 4 years (19–29 years), body weight: 73 ± 7 kg (62–87 kg), height: 1.82 ± 0.02 m, BMI: 22 ± 2 kg/m^2^) and older men (age: 74 ± 4 years (68–81 years), body weight: 79 ± 7 kg (66–92 kg), height: 1.74 ± 0.05 m, BMI: 26 ± 2 kg/m^2^). The older men had a lower height and, accordingly, higher BMI than the younger men (*p* < 0.05). Fasting concentrations of blood glucose, plasma insulin, glucagon, ratio of insulin to glucagon, HOMA-IR, ghrelin, GIP, GLP-1, PYY, total amino acids, and essential amino acids were comparable in healthy younger and older men (all *p* > 0.05; Table 1). Plasma concentrations of cysteine (younger: 0.002 ± 0.0, older: 0.06 ± 0.0 *p* < 0.001) and tryptophan (younger: 0.003 ± 0.0, older: 0.04 ± 0.0 *p* = 0.005) were higher, and of aspartic acid (younger: 0.003 ± 0.0, older: 0.002 ± 0.0 *p* < 0.001), glutamic acid (0.09 ± 0.0, older: 0.06 ± 0.0 *p* = 0.001), and serine (younger 0.09 ± 0.0, older: 0.07 ± 0.0 *p* = 0.033) were lower, in older than younger men. The study protocol was well tolerated by all subjects.

### 3.1. Glucose

**Figure 2:**
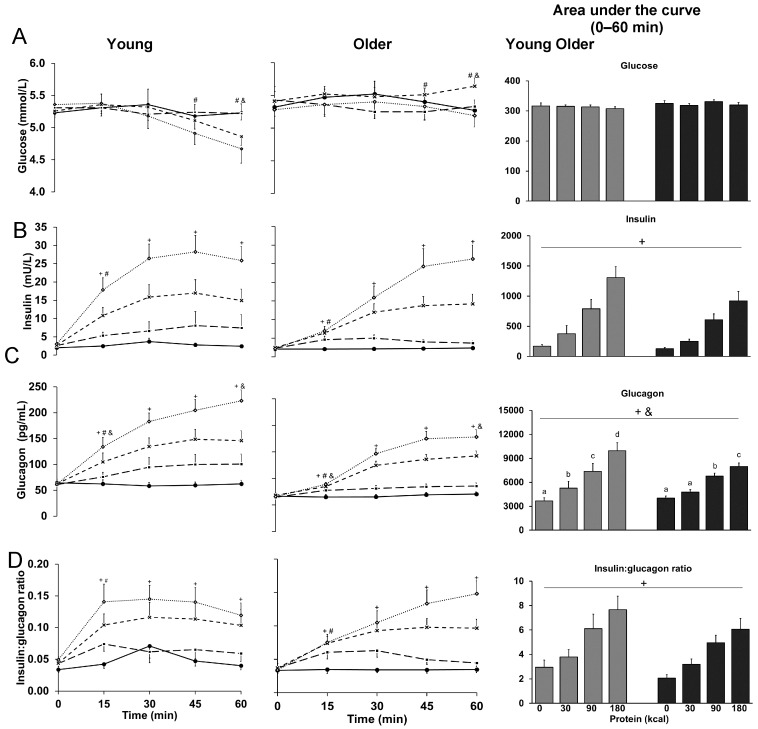
Mean (±SEM) and area under the curve concentrations of blood glucose (**A**); plasma insulin (**B**); glucagon (**C**); and ratio of insulin to glucagon (**D**) in healthy younger (*n* = 10) and older (*n* = 10) men during intraduodenal infusions of saline (control; solid line with closed circles) and whey-protein loads of 30 kcal (7.5 g protein; dashed line with closed squares), 90 kcal (22.5 g protein; dashed line with crosses) and 180 kcal (45 g protein; dotted line with open diamonds). Effects of protein-load, age, and the interaction-effect of protein-load by age were determined using a mixed-effect model with baseline concentrations as a covariate and post-hoc Bonferroni correction. Statistical significance was accepted at *p* < 0.05. + *p* < 0.05 Effect of protein-load. Post-hoc tests: higher glucagon (AUC 0–60 min and 60 min) during 30 kcal protein infusion compared to control (AUC: *p* = 0.004, 60 min: *p* = 0.029); higher insulin, glucagon, and ratio of insulin to glucagon during 90 kcal and 180 kcal protein infusions compared to control (insulin: AUC: both *p* < 0.001, 60 min: both *p* < 0.001; glucagon: AUC: both *p* < 0.001, 60 min: both *p* < 0.001; ratio of insulin to glucagon: AUC: *p* < 0.001 and *p* = 0.002, 60 min: *p* = 0.001 and *p* < 0.001). # *p* < 0.05 Effect of age. & *p* < 0.05 Interaction-effect of protein-load by age. ^a^, ^b^, ^c^, ^d^ *p* < 0.05 Interaction-effect post-hoc tests: a different letter indicates a difference between protein loads (AUC 0–60 min; ^‡^ *p* < 0.05, ^‡‡^ *p* < 0.01, ^‡‡‡^ *p* < 0.001): higher glucose in older than younger men at 60 min during 90 kcal protein infusion (^‡‡^), and in younger, but not older men, lower glucose concentrations (60 min) after the 90 kcal (^‡^) and 180 kcal (^‡^) protein infusions compared to control; lower glucagon in older than younger men at 15 min during 90 (^‡^) and 180 kcal (^‡‡^) protein infusions; in younger and older men, higher glucagon (AUC and 60 min) during 180 kcal protein infusion compared to 90 kcal (younger: ^‡‡‡^, older: ^‡^), 30 kcal (younger: ^‡‡‡^, older: ^‡‡^) and control infusions (younger: ^‡‡‡^, older: ^‡‡^), and 90 kcal protein infusion compared to 30 kcal (younger: ^‡‡^, older: *p* = ^‡^) and control infusions (younger: ^‡‡‡^, older: ^‡‡‡^); in younger (^‡^), but not older, men, higher glucagon (AUC and 60 min) during 30 kcal protein infusion compared to control.

Blood glucose concentrations at 60 min, immediately before the buffet meal, were higher in older than younger men (*p* = 0.031); glucose was lower after the 90 kcal (interaction-effect post-hoc analysis: *p* = 0.036) and 180 kcal (*p* = 0.037) protein infusions compared to control in younger, but not older men (*p* > 0.05), and higher in older than younger men after the 90 kcal protein infusion (*p* = 0.001). 15 min and AUC blood glucose concentrations were not affected by protein-load or age, with no protein-load by age (Table 1 and Figure 2).

### 3.2. Insulin

Plasma insulin concentrations at 15 min protein-load dependently increased (*p* < 0.001; Table 1 and Figure 2), and were lower in older than younger men (*p* = 0.003); insulin was higher during all protein infusions compared to control (protein-load effect post-hoc analysis: *p* < 0.05). 60 min (*p* < 0.05) and AUC (*p* < 0.05) plasma insulin concentrations protein-load dependently increased; concentrations were higher during the 90 kcal and 180 kcal protein infusions compared to control (interaction-effect post-hoc analyses: *p* < 0.001).

### 3.3. Glucagon

Plasma glucagon concentrations at 15 min protein-load dependently increased (*p* < 0.001; Table 1 and Figure 2) and were lower in older than younger men (*p* = 0.021); glucagon was lower in older than younger men during the 90 kcal (interaction-effect post-hoc analysis: *p* = 0.048) and 180 kcal (*p* = 0.008) protein infusions, higher during all protein infusions compared to control in younger men (*p* < 0.05), and higher during the 30 kcal protein infusion compared to control in older men (*p* = 0.003). 60 min (*p* < 0.001) and AUC (*p* < 0.001) plasma glucagon concentrations protein-load dependently increased in younger and older men; glucagon was higher during all protein infusions compared to control in younger men (interaction-effect post-hoc analyses: *p* < 0.001), and during the 90 kcal and 180 kcal protein infusions compared to control in older men (*p* < 0.001).

### 3.4. Ratio of Insulin to Glucagon

The ratio of plasma insulin to glucagon concentrations at 15 min protein-load dependently increased (*p* = 0.001; Table 1 and Figure 2) and was lower in older than younger men (*p* = 0.045); the ratio was higher during all protein infusions compared to control (protein-load post-hoc analyses: *p* < 0.05). The 60 min (*p* < 0.001) and AUC (*p* < 0.001) ratio of plasma insulin to glucagon concentrations protein-load dependently increased; ratios were higher during the 90 kcal (protein-load effect post-hoc analysis: *p* = 0.001) and 180 kcal (*p* = 0.05) protein infusions compared to control.

### 3.5. Ghrelin

**Figure 3:**
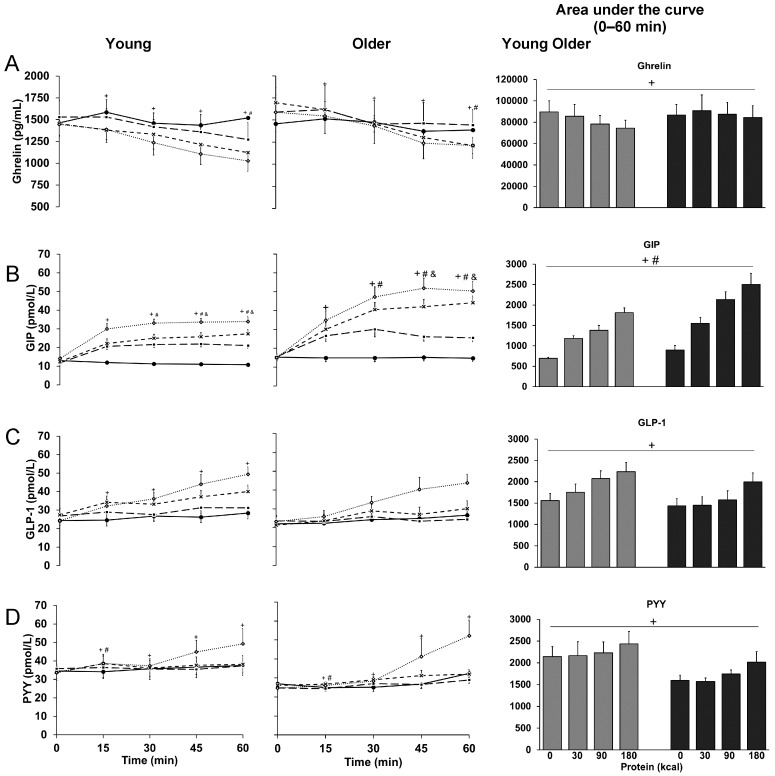
Mean (±SEM) and area under the curve concentrations of plasma ghrelin (**A**), gastric inhibitory peptide (GIP) (**B**), glucagon-like peptide-1 (GLP-1) (**C**), and peptide tyrosine-tyrosine (PYY) (**D**), in healthy younger (*n* = 10 for ghrelin and PYY; *n* = 9 for GIP and GLP-1) and older (*n* = 10) men during intraduodenal infusions of saline (control; solid line with closed circles) and whey-protein loads of 30 kcal (7.5 g protein; dashed line with closed squares), 90 kcal (22.5 g protein; dashed line with crosses), and 180 kcal (45 g protein; dotted line with open diamonds). Effect of protein-load, age, and the interaction-effect of protein-load by age were determined using a mixed-effect model with baseline concentrations as a covariate and post-hoc Bonferroni correction; statistical significance was accepted at *p* < 0.05. + *p* < 0.05 Effect of protein-load. Post-hoc tests: higher GIP (60 min, AUC 0–60 min; ^‡^ *p* < 0.05, ^‡‡^ *p* < 0.01, ^‡‡‡^ *p* < 0.001) during 30 kcal protein infusion compared to control (^‡‡‡^); lower ghrelin and higher GIP and GLP-1 during 90 kcal and 180 kcal protein infusions compared to control (ghrelin: 60 min: ^‡^, AUC: ^‡‡^; GIP: 60 min: ^‡‡‡^, AUC: ^‡‡‡^; GLP-1: 60 min, 180 kcal: ^‡‡‡^, AUC: ^‡^). # *p* < 0.05 Effect of age. & *p* < 0.05 Interaction-effect of protein-load by age. Post-hoc tests: higher GIP in older than young at 60 min during 90- (^‡‡‡^) and 180- (^‡^) kcal protein infusions.

15 min, 60 min and AUC plasma ghrelin concentrations protein-load dependently decreased (*p* < 0.05; Table 1 and Figure 3) and 60 min plasma ghrelin concentrations were higher in older than younger men *(p* = 0.029); 60 min and AUC plasma ghrelin concentrations were lower during the 90 kcal (protein-load effect post-hoc analyses; 60 min: *p* < 0.001, AUC: *p* = 0.006) and 180 kcal (*p* = 0.015, *p* = 0.002) protein infusions compared to control.

### 3.6. GIP

15 min, 60 min, and AUC plasma GIP concentrations protein-load dependently increased (*p* < 0.001; Table 1 and Figure 3) and 60 min and AUC plasma GIP concentrations were higher in older than younger men (*p* < 0.05); 60 min plasma GIP concentrations were higher in older than younger men during the 90 kcal (interaction-effect post-hoc analysis: *p* < 0.001) and 180 kcal (*p* = 0.013) protein infusions; and 15 min, 60 min, and AUC plasma GIP concentrations were higher during all protein infusions compared to control (protein-load post-hoc analyses: *p* < 0.001).

### 3.7. GLP-1

15 min, 60 min, and AUC plasma GLP-1 concentrations protein-load dependently increased (*p* < 0.05; Table 1 and Figure 3); 15 min plasma GLP-1 concentrations were higher during the 180 kcal protein infusion compared to control (protein-load post-hoc analysis: *p* = 0.014), and 60 min and AUC plasma GLP-1 concentrations were higher during the 90 kcal (60 min: *p* = 0.053, AUC: *p* = 0.034) and 180 kcal (*p* < 0.001, *p* = 0.005) protein infusions compared to control.

### 3.8. PYY

Plasma PYY concentrations at 15 min protein-load dependently increased (*p* = 0.005; Table 1 and Figure 3) and were lower in older than younger men (*p* = 0.009); PYY was higher during the 90 kcal protein infusion compared to control (protein-load post-hoc analysis: *p* = 0.005). 60 min (*p* = 0.018) and AUC (*p* = 0.042) plasma PYY concentrations protein-load dependently increased in younger and older men.

### 3.9. Amino Acids

**Figure 4:**
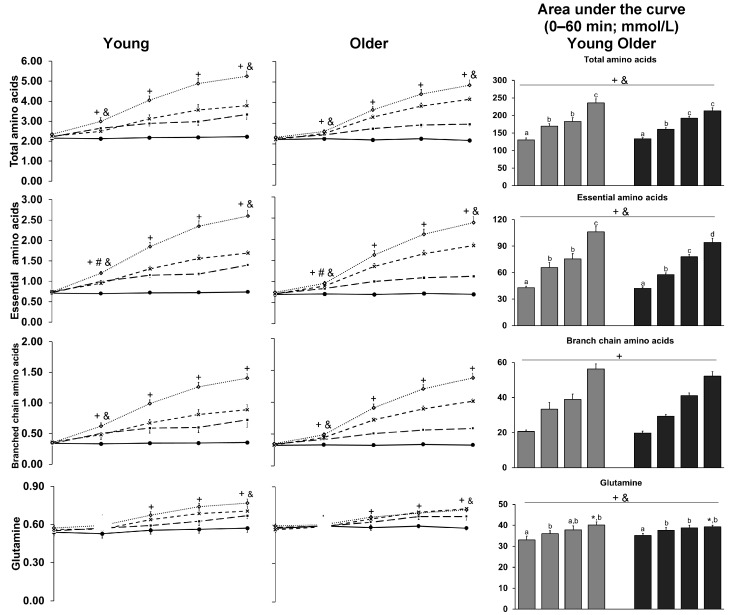

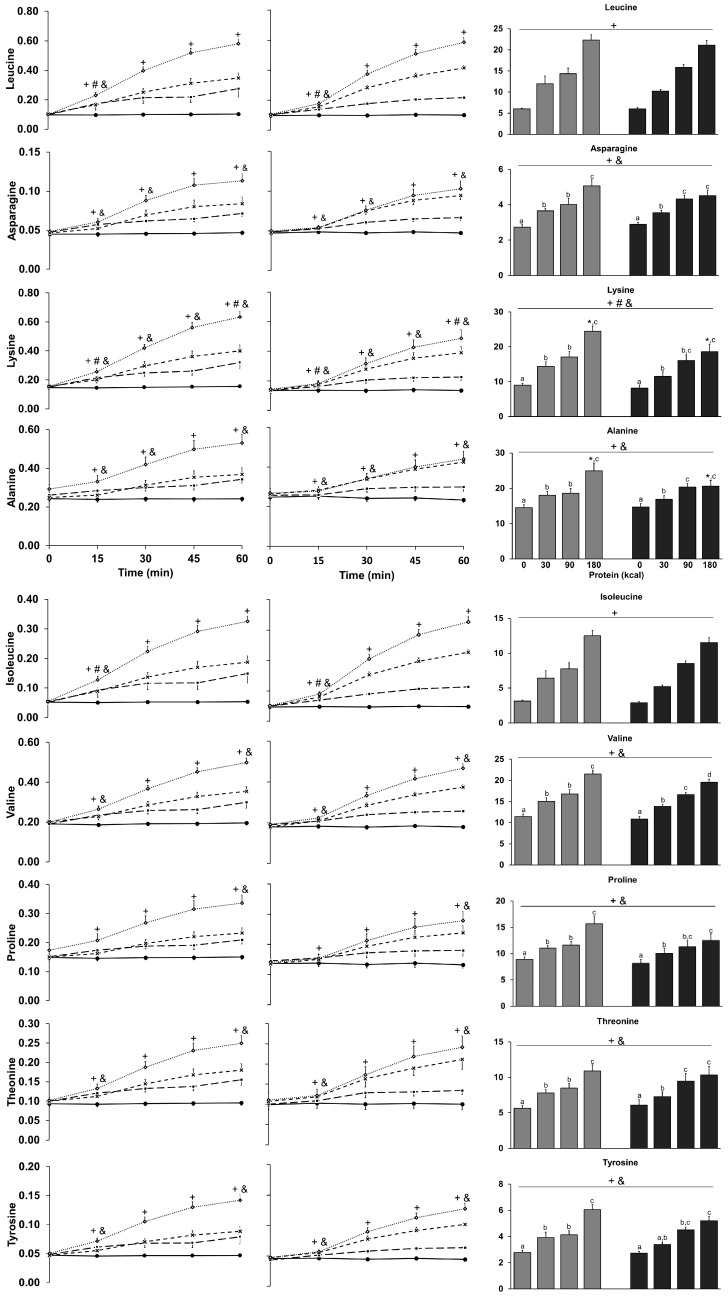

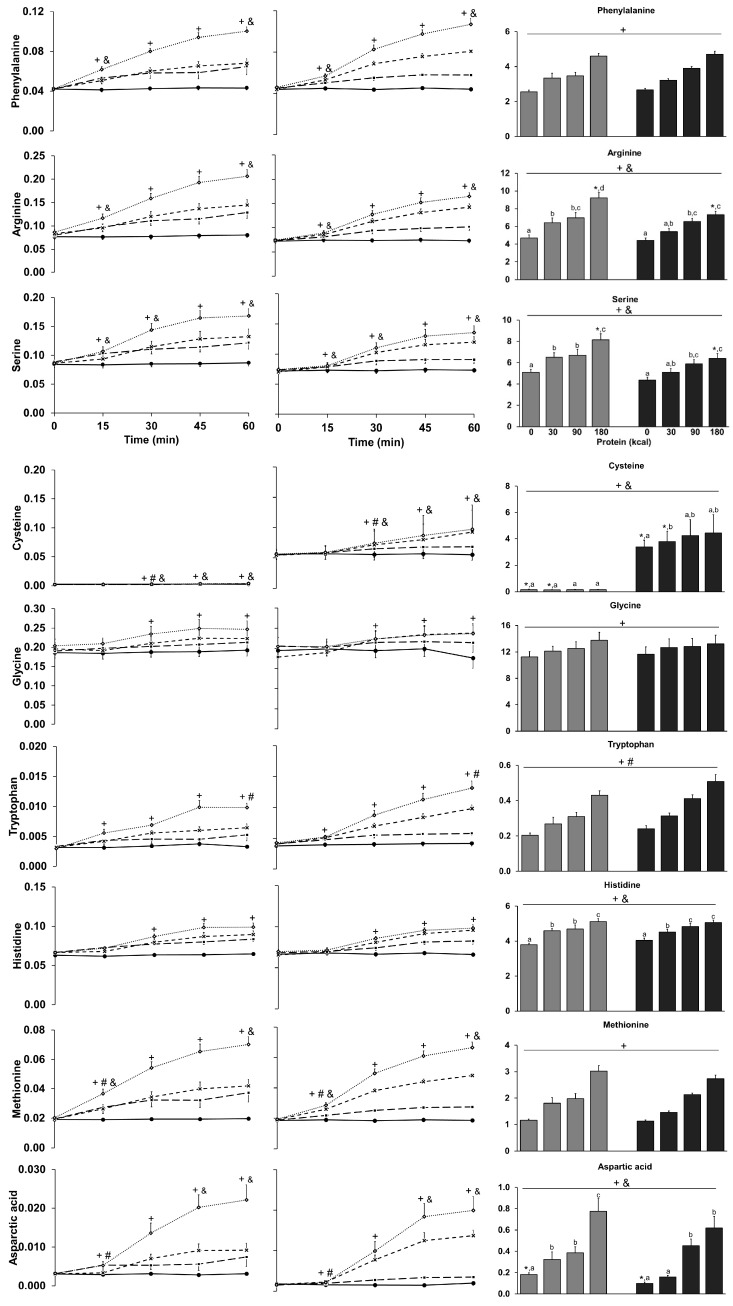

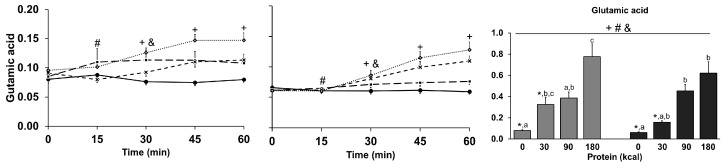
Mean (±SEM) and area under the curve concentrations of plasma total amino acids, essential amino acids (histidine, isoleucine, leucine, lysine, methionine, phenylalanine, threonine, tryptophan, and valine), branched chain amino acids (isoleucine, leucine, and valine), and individual amino acids ranked in order of highest to lowest prevalence of amino acids in the whey protein hydrolysate, in healthy younger (*n* = 10; *n* = 9 for histidine) and older (*n* = 10) men during intraduodenal infusions of saline (control; solid line with closed circles) and whey-protein loads of 30 kcal (7.5 g protein; dashed line with closed squares), 90 kcal (22.5 g protein; dashed line with crosses), and 180 kcal (45 g protein; dotted line with open diamonds). Main effect of protein-load, age, and interaction effects were determined using a mixed-effect model with baseline concentrations as a covariate and post-hoc Bonferroni correction; statistical significance was accepted at *p* < 0.05. + *p* < 0.05 Effect of protein-load. # *p* < 0.05 Effect of age. & *p* < 0.05 Interaction-effect of protein-load by age. * *p* < 0.05 Interaction-effect post-hoc tests: older compared to younger men (AUC 0–60 min); ^a^, ^b^, ^c^, ^d^ *p* < 0.05 a different letter indicates a difference between protein loads (AUC 0–60 min; ^‡^ *p* < 0.05, ^‡‡^ *p* < 0.01, ^‡‡‡^ *p* < 0.001): at 15 min during 180 kcal protein infusion, older compared to younger men: lower total (^‡^), essential (^‡^), branched chain amino acids (^‡^), leucine (^‡^), asparagine (^‡^), lysine (^‡^), isoleucine (^‡^), valine (^‡^), threonine (^‡^), tyrosine (^‡^), phenylalanine (^‡^), asparagine (^‡^), serine (^‡^), and methionine (^‡^); at 60 min: lower glutamine (^‡^), lysine (^‡^), arginine (^‡^), and higher phenylalanine (^‡^), and aspartic acid (^‡^) during 90 kcal protein infusion, and cysteine during 30 kcal protein (^‡^) and control (^‡‡^) infusion.

15 min, 60 min, and AUC plasma total amino acid concentrations protein-load dependently increased during the infusions (*p* < 0.001; Table 1 and Figure 4), and 15 min concentrations were lower in older than younger men (*p* = 0.049); plasma AUC total (interaction-effect post-hoc analysis: *p* = 0.022) and essential (*p* = 0.014) amino acid concentrations were lower during the 180 kcal protein infusion in older than younger men.

### 3.10. Relationships of Energy Intake, Gut Hormones, and Amino Acids

**Figure A1:**
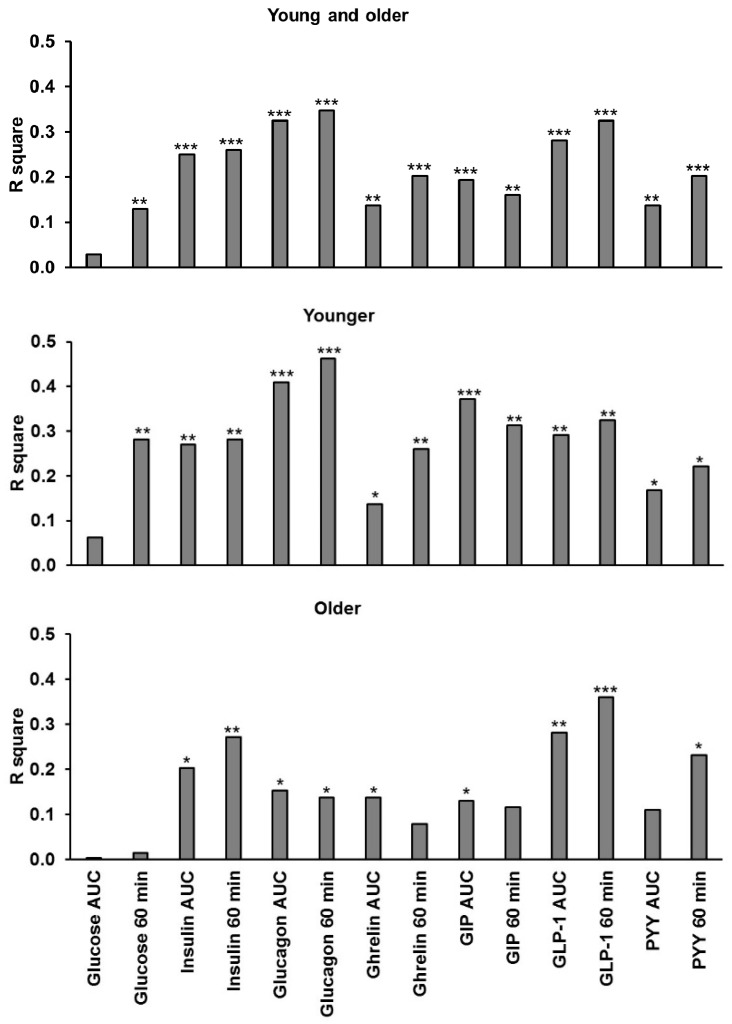
Within-subject relationships between energy intake (results of suppression of *ad libitum* energy intake after the intraduodenally infused whey protein loads have been published previously [31]) and area under the curve (AUC 0–60 min) of glucose, insulin, glucagon, ghrelin, GIP, GLP-1, and PYY concentrations in healthy older and younger men combined (*n* = 20; *n* = 19 for GIP and GLP-1), younger men (*n* = 10; *n* = 9 for GIP and GLP-1), and older men (*n* = 10). Within subject correlations were determined using a general linear model with fixed slope and random intercept. Statistical significance was accepted at *p* < 0.05. * *p* < 0.05, ** *p* < 0.005, *** *p* < 0.001.

**Figure A2:**
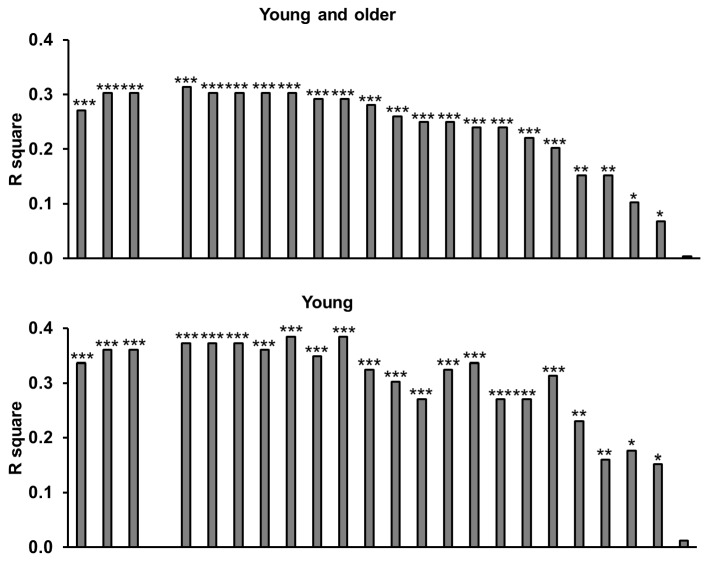

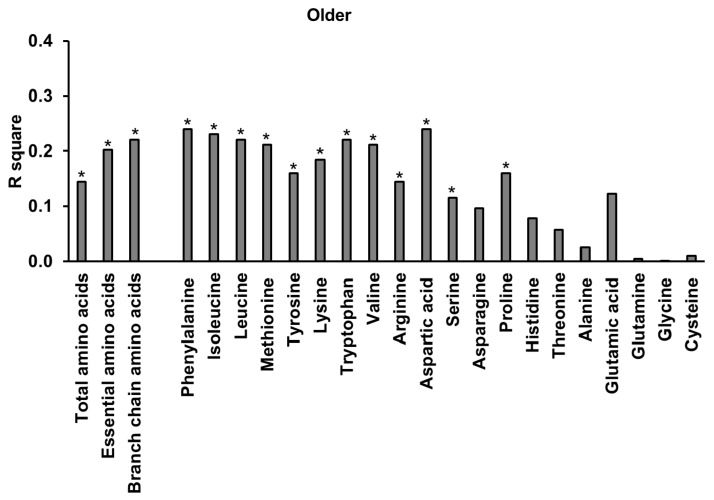
Within-subject relationships between energy intake [results of suppression of *ad libitum* energy intake after the intraduodenally infused whey-protein loads have been published previously [31]] and area under the curve (AUC 0–60 min) of total, essential amino acids (histidine, isoleucine, leucine, lysine, methionine, phenylalanine, threonine, tryptophan, and valine), branched chain amino acids (isoleucine, leucine, and valine), and individual amino acids in healthy older and younger men combined (*n* = 20; *n* = 19 for histidine), younger men (*n* = 10; *n* = 9 for histidine), and older men (*n* = 10), ranked in order of the strongest to weakest R square in the combined group. Within-subject correlations were determined using a general linear model with fixed slope and random intercept. Statistical significance was accepted at *p* < 0.05.* *p* < 0.05, ** *p* < 0.005, *** *p* < 0.001.

Energy intake (published previously [31]; younger men: control: 1270 ± 150 kcal, 30 kcal: 1123 ± 151 kcal, 90 kcal: 1028 ± 163 kcal, and 180 kcal: 851 ± 161 kcal; older men: control: 1068 ± 93 kcal, 30 kcal: 1129 ± 91 kcal, 90 kcal: 1123 ± 98 kcal and 180 kcal: 899 ± 103 kcal) was positively related to plasma ghrelin (AUC and 60 min) and glucose concentrations (60 min), and negatively related to plasma insulin, glucagon, GIP, GLP-1, and PYY concentrations (AUC and 60 min; Figure A1) and plasma amino acid concentrations (except for cysteine; Figure A2). Plasma total amino acid concentrations correlated positively with plasma insulin, glucagon, ghrelin, GIP, GLP-1, PYY, and negatively with ghrelin concentrations (AUC; Table 2).

GIP was, within subjects, related to GLP-1 (*r* = 0.68 *p* < 0.001); i.e., the greater the increase in plasma GIP concentrations, the greater the increase in GLP-1. Ghrelin was, *within subjects*, inversely related to insulin (*r* = −0.33, *p* = 0.010); i.e., the greater the increase in plasma insulin concentrations, the greater the inhibition of ghrelin production.

## 4. Discussion

The intraduodenal whey-protein infusion was at rates lower than (0.5 kcal/min), which were comparable to (1.5 kcal/min), and at the upper end (3.0 kcal/min) of normal gastric energy emptying rates [39], dose-dependently suppressed plasma concentrations of ghrelin, and stimulated concentrations of insulin, glucagon, GIP, GLP-1, PYY, and amino acids in younger, as well as older, men. Plasma concentrations of the hormones insulin, glucagon, GLP-1, and PYY, which are secreted by the small intestine in response to the presence of nutrients, and largely act to suppress appetite and food intake, were increased to a comparable degree by protein infusions in younger and older men. GIP responses were greater in older than younger men. Our observations of intraduodenally infused whey protein in older people extend the previously reported data of the acute effects of orally ingested whey protein on plasma insulin, glucagon, ghrelin, GIP, and GLP-1 concentrations in young [40,41] and older [42] adults.

Consistent with their effects on appetite, these gut hormone concentrations were negatively correlated with energy intake at the subsequent *ad libitum* meal. There was a dose-dependent suppression of energy intake by protein infusion (0, 30, 90, 180 kcal) in the younger men, and this was greater than the suppression in older men, which was only significant after the 180 kcal protein infusion [31]. In contrast, plasma concentrations of ghrelin, which is mainly secreted by the stomach and acts to stimulate appetite and food intake [43], were suppressed by the protein infusions to a comparable degree in both age groups and were positively correlated to subsequent energy intake. Plasma concentrations of amino acids correlated negatively with energy intake at the *ad libitum* meal (published previously [31]), particularly for tryptophan and tyrosine, having the highest R square in younger men, confirming previous reports [36,44,45], and lower R squares in older (range: 0.0–0.24) than younger men (range: 0.01–0.38). The higher tryptophan concentrations in younger compared to older men may have contributed to the age differences in suppression of energy intake by the intraduodendally infused whey protein.

While the younger and older men had comparable glucose, insulin, and glucagon concentrations during the 60 min protein infusions, as assessed by the area under the curve method, insulin and glucagon concentrations increased more slowly in older than younger men (e.g., significantly lower concentrations at 15 min) and glucose concentrations immediately before the buffet meal (60 min) were lower during the ‘higher’ protein-load infusions (90 and 180 kcal) compared to control in young, but not older, men. We have reported that blood glucose and insulin concentrations were higher during intraduodenal glucose infusions in healthy older than younger men [18], which is likely related to reduced insulin sensitivity with aging or a differential secretaguogue activity of glucose and amino acids on insulin-producing pancreatic beta cells. The slightly different responses in healthy older compared to younger men after whey protein administered directly into the small intestine confirms the effects of ageing on postprandial glycaemia independent of differences in insulin resistance; both age groups had comparable HOMA-IR.

Healthy older and younger men had comparable AUC ghrelin concentrations during the protein infusions, consistent with responses to mixed-nutrient intake in some [25,26] but not all previous studies [23,24,27]. It has been suggested that aging-related changes in body composition (i.e., a decrease in lean mass and increase in fat mass) may act to decrease fasting [46] and postprandial [27] ghrelin concentrations, as body fat is negatively correlated to ghrelin concentrations [47] and tends to increase with aging. Other studies, however, have found higher postprandial and fasting ghrelin concentrations in older than younger adults and impaired suppression of ghrelin after consumption of a mixed-nutrient meal in older than younger subjects [23,24]. The results of this study, where protein was infused directly into the small intestine, thereby bypassing and eliminating variations in the rate of gastric emptying, support the latter findings; plasma ghrelin levels were higher at the end of the protein infusions, immediately before the buffet meal, in older than younger men.

Plasma GIP concentrations increased rapidly during the protein infusions and these were higher in older than younger men, which may be related to differences in small intestinal transit of the whey protein. Previously, orally ingested [19,20], but not intraduodenally infused [48], glucose evoked greater GIP responses in older than younger adults.

GLP-1 and PYY are mainly secreted more distally in the gastrointestinal tract (i.e., ileum and colon) than GIP (expressed mainly in the duodenum and jejunum), and their plasma concentrations increased more slowly than GIP concentrations during the protein infusions. This was particularly so for PYY concentrations, which increased during the 180 kcal protein load largely after 30 min onwards and more convincingly in the older men. Nevertheless older and younger men had comparable plasma AUC GLP-1 and PYY concentrations during the protein infusions, consistent with responses during intraduodenal infusions of lipid and glucose [22]. Oral glucose [20] and mixed macronutrient [21] ingestion, however, are reported to increase GLP-1 concentrations more in older than younger women, again highlighting the age-related differences in hormone responses to nutrients depending on their route of delivery—older compared to younger adults have slightly slower gastric emptying [30].

The older men had a slower increase in plasma concentrations of essential amino acids (lower concentrations at 15 min after starting the whey-protein infusions), particularly leucine, isoleucine, and lysine, than the young men. Our findings are consistent with previous reports that plasma amino acid concentrations peak later and remain elevated longer after amino acid ingestion in older than younger adults, resulting in comparable AUC concentrations between age groups [9]. The infused whey protein contained 12% leucine and 2.4% glycine, amino acids which are thought to play an important role in the modulation of skeletal muscle metabolism [49,50,51]. The maximum increase in plasma amino acid concentration at the end of the protein infusion, compared to baseline, was comparable in younger and older men for most amino acids, except that the older men had higher maximum increase in aspartic acid and lower increases in alanine and glutamine than the younger men. This is an important finding, as elevated amino acid concentrations are a major determinant of muscle protein synthesis [7] and, due to suboptimal protein intake, may not always be high enough to have an optimal effect in older people. Studies utilizing stable isotope-labelled amino acids have shown that older adults have a reduced sensitivity of muscle protein synthesis to the ingestion of relatively small amounts (≤20 g) of whey protein compared to younger adults [7]. These postprandial differences between the younger and old were, however, not evident after consumption of ample amounts of dietary protein (>20 g). The higher maximum increase of asparctic acid and lower maximum increases of alanine and glutamine in older than younger men are likely the result of age-related differences in digestion, absorption, or metabolism, including, for example, higher first-pass splanchnic extraction of amino acids in in older than younger adults [52].

Plasma amino acids concentrations correlated positively with plasma concentrations of insulin, glucagon, GIP, GLP-1, and PYY and negatively with those of ghrelin. Interaction of dietary amino acids, oligopeptides, and proteins in the gut induce the so-called ileal-brake mechanism, including inhibition of proximal gastrointestinal motility, which stimulates the vagus nerve afferents to convey information to the nucleus of the solitary tract in the brainstem and thereby restricts food intake in the short term [53].

After their oral ingestion, nutrients empty from the stomach at a rate of 1–4 kcal/min [54]. Gastric emptying of nutrients is slower in older than younger adults, and in our previous study of orally administered whey protein the mean rate of gastric emptying after protein ingestion was 0.8 kcal/min in older men, compared to 1 kcal/min in younger men (*p* = 0.02) [30]. In the present study, intraduodenal whey protein suppressed subsequent voluntary energy intake in older men only when infused into the duodenum at a rate of 3 kcal/min, high in the normal range of 1–4 kcal/min, but not at lower rates of 0.5 and 1.5 kcal/min. These findings suggest that orally ingested whey protein, even in high doses, has little, if any, suppressive effect on appetite and energy intake in older people, because it cannot pass quickly enough into the small intestine to exert these effects. Consistent with this, orally ingested whey protein supplements up to 280 kcal (70 g protein) did not suppress food intake in older men in our previous study and actually increased total (drink plus meal) energy intake [30]. These findings have clinical implications. They suggest that whey protein can be ingested orally by older people at risk of, or with, malnutrition or muscle loss, in amounts high enough to have beneficial effects on their nutrition state and muscle [7], without reducing, and in fact more likely increasing, their overall energy intake. Also, the higher incretin hormone GIP response following the intraduodenal whey-protein infusions in older than younger men is likely to be beneficial for glycemic control in older people. While the plasma glutamic acid, lysine, and tryptophan concentrations during the intraduodendal whey-protein infusions were different between healthy older and younger men, total and essential amino acid concentrations, including leucine, were comparable. These results suggest that regular ingestion of whey protein in sufficient quantities is likely to have benefits in preventing muscle loss during ageing, including delaying the onset of sarcopenic obesity.

This study has several limitations, including the relatively small subject numbers. We studied only men, as they appear to have the greatest ability to respond to energy manipulation [28], so the findings require confirmation in women. Although the total loads of hydrolyzed whey protein delivered (i.e., 8, 24, and 48 g) are representative of a snack or main meal, the observed findings may be different for other protein sources.

## 5. Conclusions

Intraduodenal whey-protein infusions resulted in load-dependent changes in gut hormones and amino acids in younger and older men, and these responses were related to subsequent *ad libitum* energy intake. Plasma concentrations of insulin, glucagon, GLP-1, and PYY were increased and ghrelin decreased to a comparable degree by the infusions in both age groups, while GIP responses were greater in older than younger men.

## Figures and Tables

**Table 1:** 15 min, 60 min, and AUC blood glucose, plasma insulin, glucagon, ghrelin, GIP, GLP-1, PYY, total and essential amino acid concentrations, and the ratio of insulin to glucagon and HOMA-IR in healthy younger and older men.

		Younger				Older		
Concentrations	Baseline	15 min	60 min	AUC	Baseline	15 min	60 min	AUC
HOMA index	0.6 ± 0.1				0.5 ± 0.1			
Glucose (mmol/L)	5.3 ± 0.1	5.3 ± 0.1	5.0 ± 0.1 #&	312 ± 4	5.4 ± 0.1	5.4 ± 0.1	5.4 ± 0.1 #&	323 ± 6
Insulin (mU/L)	2.6 ± 0.4	9 ± 1 +#	13 ± 2 +	824 ± 126 +	2.3 ± 0.3	5 ± 1 +#	12 ± 2 +	593 ± 89 +
Glucagon (pg/mL)	62 ± 7	94 ± 12 +#&	133 ± 14 +&	7541 ± 836 +&	67 ± 4	79 ± 4 +#&	119 ± 7 +&	6514 ± 313 +&
Insulin to glucagon ratio	0.04 ± 0.0	0.09 ± 0.01 +#	0.10 ± 0.02 +	5.1 ± 0.7 +	0.04 ± 0.01	0.06 ± 0.01 +#	0.08 ± 0.01 +	4.1 ± 0.5 +
Ghrelin (pg/mL)	1223 ± 101	1471 ± 153 +	1235 ± 130 +#	79,470 ± 8396 +	1266 ± 120	1574 ± 207 +	1313 ± 156 +#	87,509 ± 12,176 +
GIP (pmol/L)	13 ± 1	19 ± 3 +	23 ± 1 +#&	1458 ± 83 +#	15 ± 2	27 ± 3 +	34 ± 3 +#&	2063 ± 185 +#
GLP-1 (pmol/L)	26 ± 3	30 ± 3 +	37 ± 3 +	2023 ± 169 +	23 ± 3	24 ± 3 +	31 ± 4 +	1675 ± 189 +
PYY (pmol/L)	34 ± 4	37 ± 4 +#	41 ± 5 +	2276 ± 265 +	26 ± 1	26 ± 1 +#	36 ± 3 +	1776 ± 115 +
Total amino acids (mmol/L)	2.2 ± 0.01	2.6 ± 0.2 +&	3.6 ± 0.2 +&	236 ± 12 +&	2.3 ± 0.01	2.4 ± 0.1 +&	3.5 ± 0.1 +&	225 ± 7 +&
Essential amino acids (mmol/L)	0.7 ± 0.01	1.0 ± 0.04 +#&	1.61 ± 0.13 +#	106 ± 6 +&	0.7 ± 0.01	0.8 ± 0.02 +#&	1.52 ± 0.1 +#	94 ± 5 +&

Mean (±SEM) for 15 min, 60 min, and area under the curve (AUC) blood glucose, plasma insulin, glucagon, ghrelin, gastric inhibitory peptide (GIP), glucagon-like peptide-1 (GLP-1), peptide tyrosine-tyrosine (PYY), total and essential amino acids concentrations, and the ratio of insulin to glucagon and Homeostatic model assessment (HOMA) index in healthy younger (*n* = 10, *n* = 9 for GIP and GLP-1) and older (*n* = 10) men during intraduodenal infusions of saline (control) and whey protein loads of 30 kcal (7.5 g protein), 90 kcal (22.5 g protein), or 180 kcal (45 g protein). Main effect of protein load, age, and interaction effects were determined using a mixed-effect model with baseline concentrations as a covariate and post-hoc Bonferroni correction; statistical significance was accepted at *p* < 0.05. + *p* < 0.05 Effect of protein load. # *p* < 0.05 Effect of age. & *p* < 0.05 Interaction-effect of protein load by age.

**Table 2:** Within-subject correlations between concentrations of total amino acids and glucose, insulin, glucagon, ghrelin, GIP, GLP-1, and PYY.

Concentrations	*r*	*p*
Glucose (mmol/L)	−0.12	0.35
Insulin (mU/L)	0.80	<0.001
Glucagon (pg/mL)	0.84	<0.001
Ghrelin (pg/mL)	−0.32	0.013
GIP (pmol/L)	0.82	<0.001
GLP-1 (pmol/L)	0.62	<0.001
PYY (pmol/L)	0.45	<0.001

*r* and *p* values of within-subject correlations between plasma concentrations of total amino acids (mmol/L; AUC) and concentrations (AUC) of blood glucose and plasma insulin, glucagon, ghrelin, gastric inhibitory peptide (GIP), glucagon-like peptide-1 (GLP-1), and peptide tyrosine-tyrosine (PYY) in younger and older men. Within-subject correlations were determined by using a general linear model with fixed slope and random intercept. *n* = 20 (*n* = 19 for plasma concentrations of GIP and GLP-1).
